# The conservation value of elevation data accuracy and model sophistication in reserve design under sea‐level rise

**DOI:** 10.1002/ece3.1669

**Published:** 2015-09-18

**Authors:** Mingjian Zhu, Tom Hoctor, Mike Volk, Kathryn Frank, Anna Linhoss

**Affiliations:** ^1^ Department of Urban PlanningBeijing Jiaotong UniversityBeijing100044China; ^2^ Department of Landscape ArchitectureUniversity of FloridaGainesvilleFlorida32611; ^3^ Department of Urban and Regional PlanningUniversity of FloridaGainesvilleFlorida32611; ^4^ Department of Agricultural and Biological EngineeringMississippi State UniversityStarkvilleMississippi39762

**Keywords:** Adaptive reserve design, biodiversity conservation, conservation priorities, Florida, sea‐level rise

## Abstract

Many studies have explored the value of using more sophisticated coastal impact models and higher resolution elevation data in sea‐level rise (SLR) adaptation planning. However, we know little about to what extent the improved models and data could actually lead to better conservation outcomes under SLR. This is important to know because high‐resolution data are likely to not be available in some data‐poor coastal areas in the world and running more complicated coastal impact models is relatively time‐consuming, expensive, and requires assistance by qualified experts and technicians. We address this research question in the context of identifying conservation priorities in response to SLR. Specifically, we investigated the conservation value of using more accurate light detection and ranging (Lidar)‐based digital elevation data and process‐based coastal land‐cover change models (Sea Level Affecting Marshes Model, SLAMM) to identify conservation priorities versus simple “bathtub” models based on the relatively coarse National Elevation Dataset (NED) in a coastal region of northeast Florida. We compared conservation outcomes identified by reserve design software (Zonation) using three different model dataset combinations (Bathtub–NED, Bathtub–Lidar, and SLAMM–Lidar). The comparisons show that the conservation priorities are significantly different with different combinations of coastal impact models and elevation dataset inputs. The research suggests that it is valuable to invest in more accurate coastal impact models and elevation datasets in SLR adaptive conservation planning because this model–dataset combination could improve conservation outcomes under SLR. Less accurate coastal impact models, including ones created using coarser Digital Elevation Model (DEM) data can still be useful when better data and models are not available or feasible, but results need to be appropriately assessed and communicated. A future research priority is to investigate how conservation priorities may vary among different SLR scenarios when different combinations of model‐data inputs are used.

## Introduction

It is important to accurately identify and delineate conservation priorities as a means of guiding future land‐use planning and decision‐making, and maintaining critical ecosystem services and green infrastructure. However, sea‐level rise (SLR) and other effects of global climate change produce a decision‐making environment marked by high uncertainty (Noss 2011). Uncertainty exists around the appropriate choice of impact assessment methods with different models and parameterizations potentially producing different results (Mcleod et al. 2010). Approaches for modeling the impacts of SLR on coastal ecosystems vary in their level of accuracy and data requirements, from simplistic “bathtub” model that assumes everything under a certain level of SLR will be inundated, to more sophisticated coastal impact models such as Sea Level Affecting Marshes Model (SLAMM; Clough et al. 2010) that account for hydrological and ecological processes (Mcleod et al. 2010).

High‐resolution elevation data (Lidar, light detection and ranging) and sophisticated coastal impact models (SLAMM) are preferably used in SLR adaptation planning because the modeling results will provide foundations for subsequent analysis relevant to SLR (Aiello‐Lammens et al. 2011; Runting et al. 2013; Linhoss et al. 2015). However, we know little about whether the improved model–dataset combination would actually lead to more efficient conservation outcomes and to what extent this model–dataset combination could improve SLR adaptive conservation outcomes. This is important to know because high‐resolution data are likely to not be available in some data‐poor coastal areas and running more complicated models is relatively time‐consuming, expensive and needs additional expert knowledge.

A recent study examining the cost‐effectiveness of different model–dataset combinations in coastal South East Queensland, Australia, showed that it is considerably more cost‐effective to use a process‐based model and high‐resolution elevation data in conservation planning for SLR, even if this requires a substantial proportion of the project budget to be spent. The less accurate model and dataset failed to identify areas of high conservation value, reducing the cost‐effectiveness of the resultant conservation plan (Runting et al. 2013). As Runting et al. (2013) pointed out that a future research priority is to quantify how the project findings vary among different regions because the findings may not hold in all situations, particularly those areas with consistently low topographic relief or different economic context. This research investigates the differences among conservation priorities produced by different model–dataset combinations in an area with consistently low topographic relief in Florida.

## Materials and Methods

### Study area

**Figure 1:**
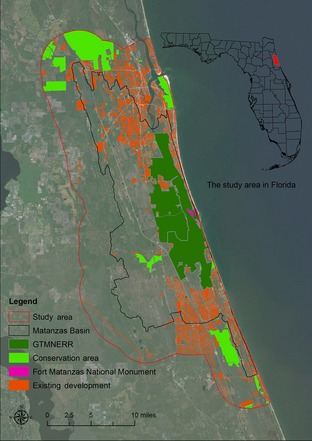
The Matanzas study area includes a 5‐km buffer beyond the Matanzas River Basin in northeast Florida to include regional ecological considerations.

The ecological and socioeconomic features of the Matanzas River Basin in Florida provide a salient case study for developing conservation designs in response to SLR. Located along northeast Florida's Atlantic coastline, the Matanzas River Basin is one of the most valued and threatened areas along the Florida coastline. The Matanzas River is a coastal estuarine water body that extends from the St. Augustine Inlet southward into the Intracoastal Waterway south to Palm Coast, Florida. The basin covers approximately 100,000 acres between the City of St. Augustine and the City of Palm Coast and a large area of rural lands to the west. The Matanzas River Basin had nearly 90% of its land in undeveloped natural or rural condition, thus providing a rare opportunity to incorporate SLR into future conservation and land‐use plans with little conflict with existing development. The basin includes the Matanzas River estuary and associated coastal forests, marshes, wetlands, uplands, and beaches. The basin contains the southern component of the Guana Tolomato Matanzas National Estuary Research Reserve (GTMNERR), the Fort Matanzas National Monument, and other Florida conservation management areas including the Matanzas State Forest and the Faver‐Dykes State Park. To incorporate more regional land‐use and ecological considerations (e.g., the forests adjacent to the west side of the basin are within the Florida Ecological Greenways Network, the study area includes a 5‐km buffer beyond the Matanzas River Basin (Fig. 1).

### Sea‐level rise scenario

Sea‐level rise scenarios are fundamental to vulnerability assessments and all other following parts of the reserve design process. To compare the conservation outcomes of different model–dataset combinations, a 1.0‐m SLR projection by 2100 is used to predict the impacts of SLR on coastal habitats in the Matanzas study area. We chose the 1.0 m SLR scenario for several reasons. First, the 1.0‐m SLR projection by the end of the 21st century is the “likely” worst‐case scenario according to the fifth assessment report of IPCC (Church et al. 2013). Second, it is likely not necessary to include different SLR scenarios in this analysis as the aim of this study was to examine conservation outcomes of different model–dataset combinations although this analysis might be useful in the future because differences in predictions and identified priority areas might be significantly different under different SLR levels (Cooper et al. 2013; Gesch 2013). Third, the 1.0‐m SLR projection has been used in a previous similar study to compare lands vulnerable to inundation by SLR with different elevation datasets (Gesch 2009). A future research priority is to investigate how conservation priorities may vary among different SLR scenarios when different combinations of data inputs are used.

### Model–dataset combinations

Using different coastal impact models and different elevation datasets can result in different conservation outcomes. To compare conservation priorities with different model–dataset combinations, two coastal impact models including SLAMM and bathtub models and two elevation datasets including Lidar‐based elevation data and the National Elevation Dataset (NED; Gesch 2007) were selected for comparison. SLAMM is a “complex” coastal impact model that simulates the geomorphological processes that result in coastal wetland conversions and shoreline modifications during long‐term SLR (Clough et al. 2010). For this study, the model was run in a participatory setting where involvement and feedback from the academic study team as well as GTMNERR personnel was integral to determining model parameters and interpreting the results (Linhoss et al. 2015). The elevation data used in this study was a composite from St. Johns County (2008), Palm Coast (2008), and FWC‐FWRI (2009) including the best available Lidar and inland elevation data. The land‐cover data used in this study was also a composite dataset that incorporates data from the St. Johns River Water Management District Land Use and Cover dataset (SJRWMD 2004), and a more detailed emergent vegetation dataset obtained from the GTMNERR (Kinser et al. 2007). Land‐cover classifications from both datasets were cross‐walked to SLAMM land‐cover classifications and converted to a 10‐m grid. Tidal range was determined from the NOAA Ft. Matanzas, Matanzas River station (Station ID 8720686). Historic SLR data were obtained from the NOAA Fernandina Beach station (Station ID 8720030). The accretion and erosion rates were parameterized from Callaway et al. (1997), NWF (2006), Craft et al. (2008), and Clough et al. (2010).

A bathtub model is a “simplistic” model that works by identifying lands under a given contour as areas inundated by a given rise in sea levels. In addition to the coastal impact models, the quality of elevation data as input data is another important variable, which affects the distribution and types of conservation outcomes identified by models such as Zonation (Moilanen et al., 2009) in response to SLR. Compared to NED, Lidar‐based elevation data have significantly better spatial resolution and vertical accuracy (Gesch 2009). In recent years, high quality elevation data derived from Lidar have become available and this “fine” resolution dataset is highly suitable for detailed study related to SLR (Gesch 2009). For this study, “fine” resolution data was a composite from St. Johns County Lidar (2008), Palm Coast Lidar (2008), and the Florida Fish and Wildlife Research Institute (Linhoss et al. 2015). The “coarse” data were NED from United States Geological Survey. The two models and datasets can be used to form four different model–dataset combinations including SLAMM–Lidar, SLAMM–NED, Bathtub–Lidar, and Bathtub–NED. For this study, the SLAMM–NED combination was not included in the analysis due to funding and timing constraints that SLAMM was only run with the Lidar elevation dataset (Linhoss et al. 2015). For this research, three model–dataset combinations were used to identify SLR adaptive conservation priorities in the Matanzas study area (Table 1).

**Table 1:** Model–dataset combinations

Model–dataset combination	Coastal impact model	Elevation dataset	Description
SLAM–Lidar	SLAMM	Lidar (10 m)	The best available DEM data with process‐based model can predict coastal habitat change in response to SLR based on key ecological processes and abiotic factors
Bathtub–Lidar	Bathtub	Lidar (10 m)	The best available DEM data with simple bathtub inundation analysis. Sites below the given elevation will be inundated
Bathtub–NED	Bathtub	NED (30 m)	The coarse input data with simple bathtub inundation analysis. This is the cheapest and fastest option, but omits key ecological processes such as hydrological process

### Comparison of conservation priorities with different model–dataset combinations

**Figure 2:**
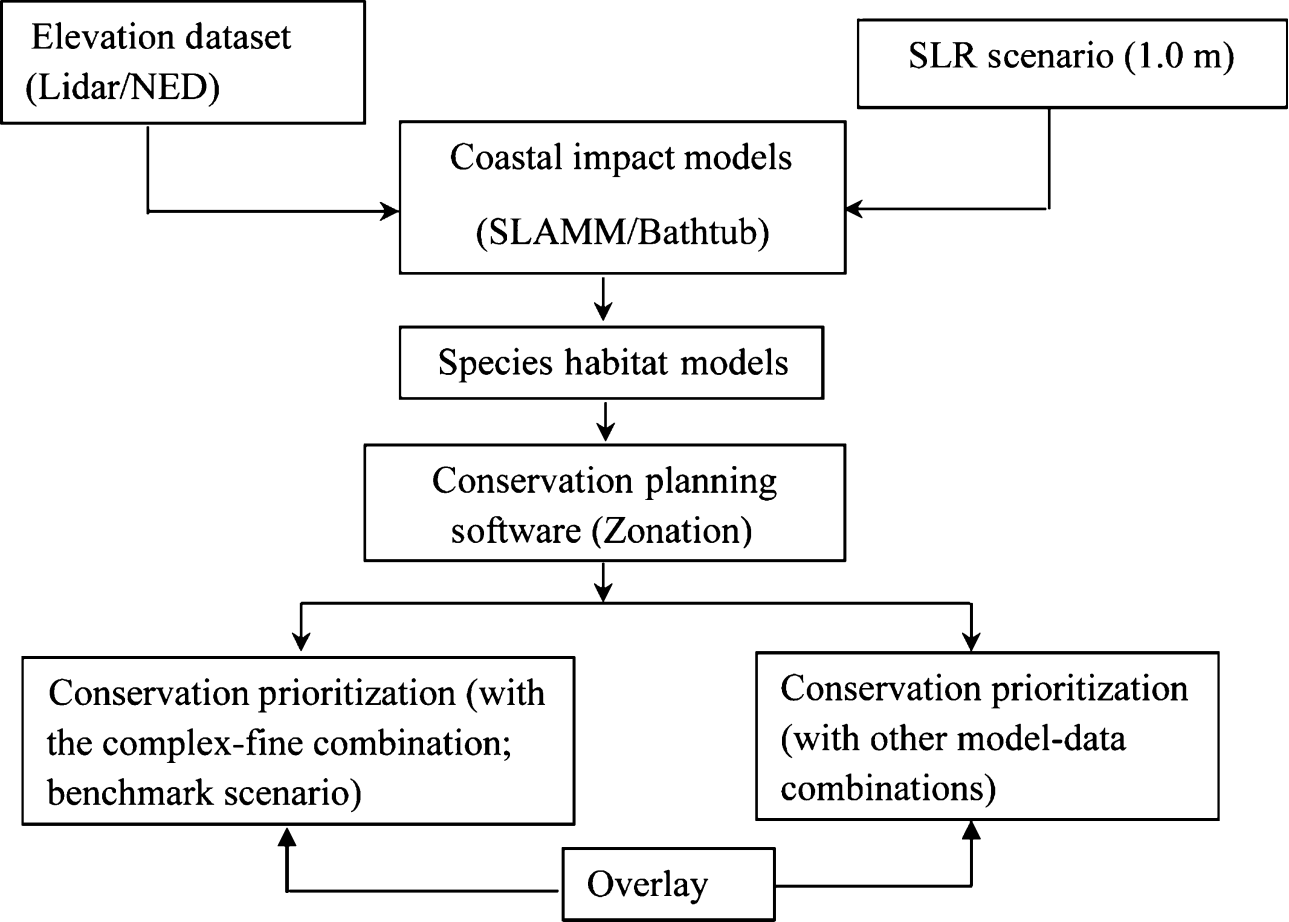
Diagram of the methodology used to compare conservation outcomes with different model–dataset combinations.

The overall methodology used in this research to compare the conservation outcomes with different model–dataset combinations is shown in Figure 2. Based on the three model–dataset combinations (SLAMM–Lidar, Bathtub–Lidar, and Bathtub–NED), three different land‐use datasets were generated as the key input to run species habitat models for selected focal species in the Matanzas study area. Using the land‐use and land‐cover grids as the primary input for species habitat models, potential species habitat in response to SLR was identified for 5 selected focal species in the Matanzas study area to demonstrate the impact of model–dataset combinations on species habitat identification. Outputs of the species habitat models were used as primary inputs for running conservation planning software Zonation to identify conservation priorities for 1.0 m SLR. The SLAMM–Lidar combination is set to be the benchmark scenario that the other two model–dataset combinations were compared in order to measure the differences of conservation outcomes with different model–dataset combinations.

### Species habitat with different model–dataset combinations

Five focal species including black rail (*Laterallus jamaicensis*), limpkin (*Aramus guarauna*), painted bunting (*Passerina ciris*), American oystercatcher (*Haematopus palliatus*), and Marian's marsh wren (*Cistothorus palustris griseus*) were selected as representative species in this study. Habitat for each of these species is considered likely to be significantly affected by future SLR. With species habitat models obtained from the Center for Landscape Conservation Planning at the University of Florida (Hoctor 2011), the Florida Fish and Wildlife Conservation Commission (FWC) and the Florida Natural Areas Inventory (FNAI), as well as species occurrence data from FNAI, current species habitat distribution and potential future species habitat distribution under the 1.0 m SLR scenario was identified. These species habitat models were developed specifically for focal species in Florida in collaboration with specific species experts and have been peer‐reviewed (Hoctor 2011).

To identify current potential habitat for focal species, a composite land‐use dataset was created that incorporates the most recent land‐use data from the St. Johns River Water Management District (SJRWMD), the Cooperative Land Cover dataset (Knight et al. 2010), and emergent vegetation data from GTM NERR as the primary data inputs with ancillary environmental data from the Florida Geographic Data Library (http://www.fgdl.org/metadataexplorer/explorer.jsp) including soils, streams, ditches, eagle nests. To identify future potential species habitat with different model–dataset combinations, three different land‐use datasets were created incorporating future land‐cover changes identified by each model–dataset combinations. The SLAMM codes were crosswalked to Florida Land Use, Cover and Forms Classification System (FLUCCS) codes (Appendix), and the inundation areas identified by the bathtub model were given a value of 5000 as estuarine water.

### Conservation prioritization using Zonation

Zonation software (Moilanen et al. 2009) was developed for spatial conservation prioritization based on observed or predicted distribution of biodiversity features (e.g., species, habitat types). This software has been used to identify conservation priorities in response to SLR in a coastal region of South East Queensland, Australia (Runting et al. 2013). Zonation can be used to identify areas important for retaining both habitat quality and connectivity for multiple species or other biodiversity features, thus providing conservation and land‐use decision makers a quantitative method to protect biodiversity in the long run (Moilanen et al. 2012). Typically, Zonation can be used to (1) assess existing and proposed conservation areas, (2) expand existing conservation areas, and (3) identify new conservation areas to achieve certain conservation goals. The primary output of Zonation is a landscape map that showing the ranking of conservation value in the study area.

For this research, we used the “core‐area Zonation” rule for identifying conservation priorities in the Matanzas study area as this rule is most appropriate when trade‐offs between species are discouraged (Moilanen et al. 2005, 2012). In addition, “distribution smoothing” was used as the aggregation method in this research as this method can effectively identify important contiguous areas where the species has high probabilities of occurrence (Moilanen et al. 2012). Using species habitat for the five selected focal species with each model–dataset combination as the inputs to run Zonation, three unique outputs identifying potential future conservation priorities can be identified.

## Results

### Land‐cover impacts with different model–dataset combinations

**Figure 3:**
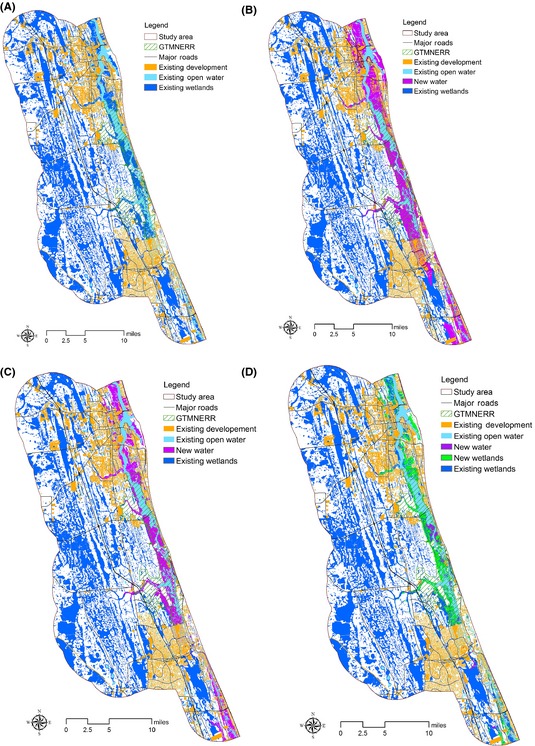
Water and wetlands distribution under (A) current conditions, (B) Bathtub–NED combination, (C) Bathtub–Lidar combination, and (D) SLAMM–Lidar combination.

Conservation planners and reserve managers responsible for mitigating SLR effects will need accurate estimation of land‐cover types to be affected by potential SLR in order to identify conservation impacts and priorities. To demonstrate SLR impacts on land‐cover types with different model–dataset combinations, land‐cover maps showing water and wetland distribution with a SLR of 1.0 m were created using all three methods previously described (Fig. 3). Compared with current land‐cover, wetland areas under a 1.0 m SLR scenario are likely to decrease with the Bathtub–NED and Bathtub–Lidar combinations but increase with the SLAMM–Lidar combinations. This is because the bathtub model can only indicate potential inundation of existing wetlands and other terrestrial land cover and not the potential conversion of uplands to wetlands that is predicted to occur during SLR. Compared with current land cover, areas of open water in the 1.0 m SLR scenario were projected to increase significantly with all the three model–dataset combinations (Table 2). The increase in the acreage of open water is due to large areas being inundated by the rising sea levels and exacerbated by the low topographic relief in the study area, especially when using the bathtub model. The results show that using different model–dataset combinations in predicting land‐cover change under SLR will clearly lead to differences in the estimated area of projected future land‐cover types; therefore, habitat delineation for focal species could also be significantly affected.

**Table 2:** Estimated areas of wetlands and water distribution under different model–dataset combinations

	Current	Bathtub–NED	Bathtub–Lidar	SLAMM–Lidar
Wetlands (acres)	89,747.21	71,779.61	73,024.75	92,056.44
Water (acres)	12,703.86	41,836.78	30,256.85	20,149.28

### Species habitat with different model–dataset combinations

**Figure 4:**
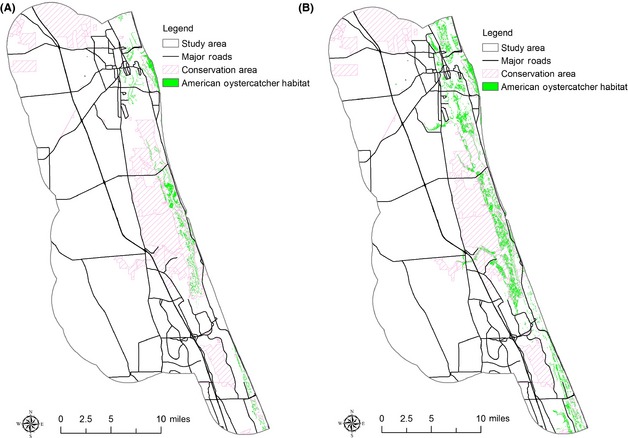
American oystercatcher habitat under (A) current conditions and (B) SLAMM–Lidar combination. No suitable habitat for American oystercatcher is identified under the Bathtub–NED and Bathtub–Lidar combinations.

Using species habitat models from the University of Florida's Center for Landscape Conservation Planning and FWC, four species habitat maps including current species habitat and species habitat under the three different model–dataset combinations were created for the five selected species (see Fig. 4 showing species habitat for American Oystercatcher as an example). Statistics describing the habitat model outputs for each species show that the use of different model–dataset combinations for predicting species habitat under SLR will clearly lead to the differences in the estimated areas of species habitat (Table 3).

**Table 3:** Areas of species habitat under different model–dataset combinations

Species	Current (acres)	Bathtub–NED (acres)	Bathtub–Lidar (acres)	SLAMM–Lidar (acres)
Black rail	1601.00	938.51	943.18	679.50
Limpkin	6003.00	4863.89	5178.28	5264.44
Painted bunting	9463.00	7144.28	7952.15	8962.44
Oystercatcher	3444.40	0	0	9207.88
Marsh Wren	6792.41	0	0	5551.20

Black rails are secretive marsh birds and this bird is usually found in high salt marsh and some freshwater marshes where soils are inundated or have only extremely shallow water (Cox and Kautz 2000). The habitat model identifies the higher areas of salt marsh that are within 300 m of uplands and more than 100 m further away from open water that black rail appear to prefer (Hoctor 2011). Compared to black rail current habitat, all the three model–dataset combinations identify much less habitat under the 1.0 m SLR scenario. This is because higher salt marsh further away from water is decreasing since open water increases as SLR. Compared to black rail habitat identified in the SLAMM–Lidar combination, habitat identified using the bathtub model is larger. This is because SLAMM identifies more wetlands but less open water than the bathtub model.

Limpkins are found in a variety of freshwater wetlands and water bodies in Florida. They usually occur in freshwater wetlands, where apple snails and other snails appear. They are also found near lake, river and stream edges, and wetlands associated with open water and herbaceous freshwater wetlands (Cox 1994). The habitat model identifies all freshwater wetlands adjacent to appropriate open water and all freshwater herbaceous wetlands as limpkin habitat (Hoctor 2011). Compared to limpkin's current habitat, all three model–dataset combinations identify fewer habitats under the 1.0 m SLR scenario. This is because freshwater will decrease as SLRs in all the three model–dataset combinations. The SLAMM–Lidar combination could identify more limpkin habitat than the other two combinations because the SLAMM model takes wetland conversions into account while the bathtub model does not, although SLAMM may still under‐represent conversion of uplands to freshwater marsh because SLAMM does not take upland‐freshwater marsh into account. The Bathtub–NED combination identifies the least limpkin habitat because a large number of freshwater wetlands are likely to be inundated in this scenario.

Painted bunting is usually found in xeric oak scrub, shrub, and brush lands in coastal areas. This species breeds in maritime hammocks or scrubby land cover (Lowther et al. 1999). The habitat model identifies all xeric oak scrub, shrub, and brush land, and then hardwood hammocks and forest and cabbage palm–live oak hammocks that were located on “extremely well”, “well”, or moderately well‐drained soils within 60 m of the xeric oak and brush land classes as potential habitat (Hoctor 2011). Compared to painted bunting's current habitat, all the three model–dataset combinations identify fewer habitats. This is because the appropriate habitat for this species will decrease as SLRs of 1.0 m in the future because it is exclusively dependent on drier upland sites. The SLAMM–Lidar combination identifies more painted bunting habitat than the other two combinations because it provides a higher resolution depiction of upland loss than bathtub models. Habitat for American oystercatcher includes shell bars, spoil islands, coastal beaches, mudflats, and salt marshes, and this species prefers large sand areas with sparse vegetation for nesting (Lauro and Burger 1989). The habitat model identifies nonvegetated wetlands, tidal flats, shorelines, oyster bars, beaches other than swimming beaches and exposed rock with marsh grasses where it has been found to occur as appropriate American oystercatcher habitat (Hoctor 2011). Compared to American oystercatcher's current habitat, the SLAMM–Lidar combination scenario identifies much more additional habitat, but the Bathtub–NED and Bathtub–Lidar combinations identify no habitat for this species. This is because the appropriate American oystercatcher habitat which includes nonvegetated wetlands, tidal flats, and other habitats is projected as inundated by the rising sea water based on simplistic bathtub models. While using SLAMM, appropriate American oystercatcher habitat can be identified because this coastal impact model recognizes wetland conversions and less inundation as SLRs comparing to the bathtub model.

Habitat for Marian's marsh wren includes tidal marshes, especially salt marshes adjacent to tidal creeks with little or no mangrove encroachment (Rogers et al. 1997). The habitat model identifies saltwater marsh within 150 m of tidal creeks as primary habitat and saltwater marsh over 150 m from tidal creeks as secondary habitat for Marian's marsh wren (Hoctor 2011). Compared to Marian's marsh wren's current habitat, the SLAMM–Lidar combination scenario identifies 18.27% less habitat, and the Bathtub–NED and Bathtub–Lidar combinations identify no habitat for this species. This is because saltwater marshes within/over 150 m of tidal creeks will be all inundated by the 1.0 m SLR based on the bathtub model, but most of this habitat will remain unaffected and some new habitat will be gained based on the SLAMM analysis.

The selected species habitat analysis demonstrated that using different model–dataset combinations could clearly lead to different outcomes of species habitat in response to SLR. Species habitat identified based on SLAMM could be much more than species habitat identified based on the bathtub model for estuarine species such as American oystercatcher and Marian's marsh wren. The results show that appropriate selection of coastal impact model and elevation dataset is very important in identifying species habitat under SLR and using the less sophisticated coastal impact model and less accurate elevation dataset could fail to identify a large number of appropriate habitats for some species.

### Conservation prioritization with different model–dataset combinations

**Figure 5:**
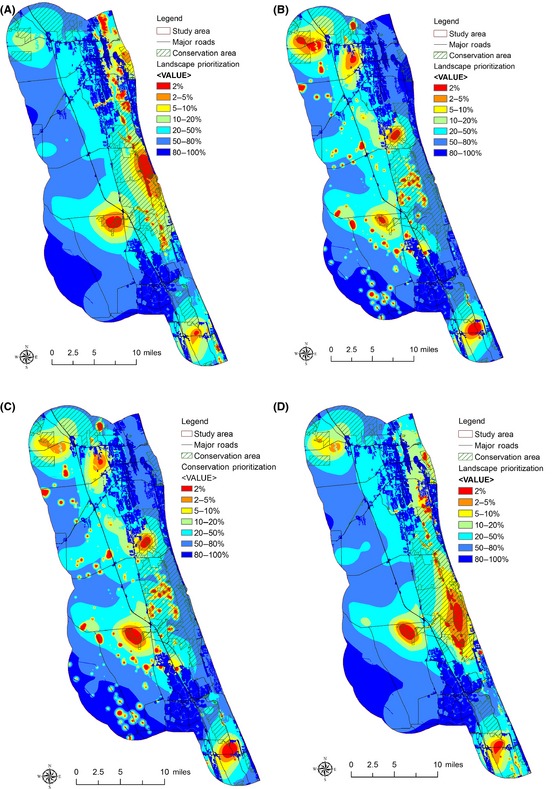
Conservation prioritization under (A) current condition (B) Bathtub–NED combination, (C) Bathtub–Lidar combination, (D) SLAMM–Lidar combination. The red color represents the top 2% conservation priority areas in the study area.

**Figure 6:**
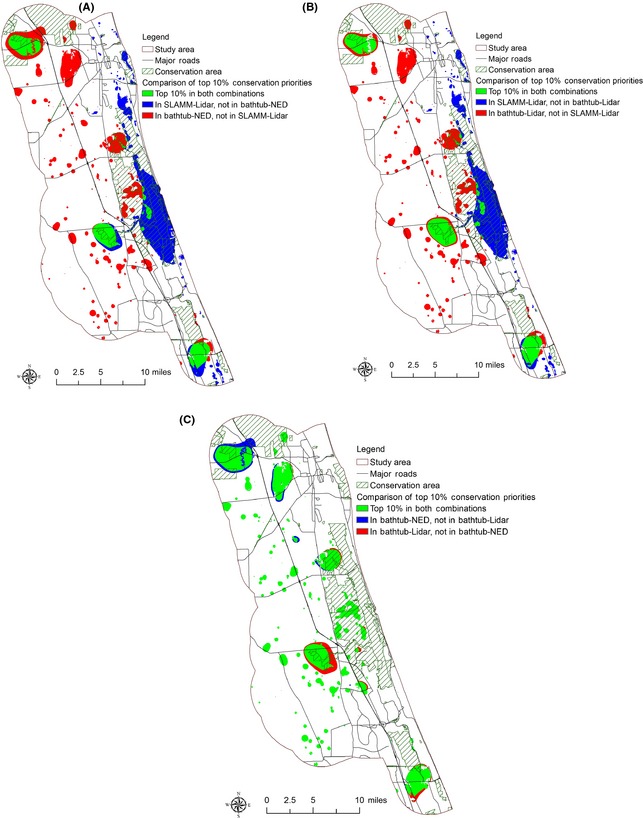
Comparison of top 10% conservation priorities identified in (A) SLAMM–Lidar combination and Bathtub–NED combination, (B) SLAMM–Lidar combination and Bathtub–Lidar combination, (C) Bathtub–NED and Bathtub–Lidar combination.

**Figure 7:**
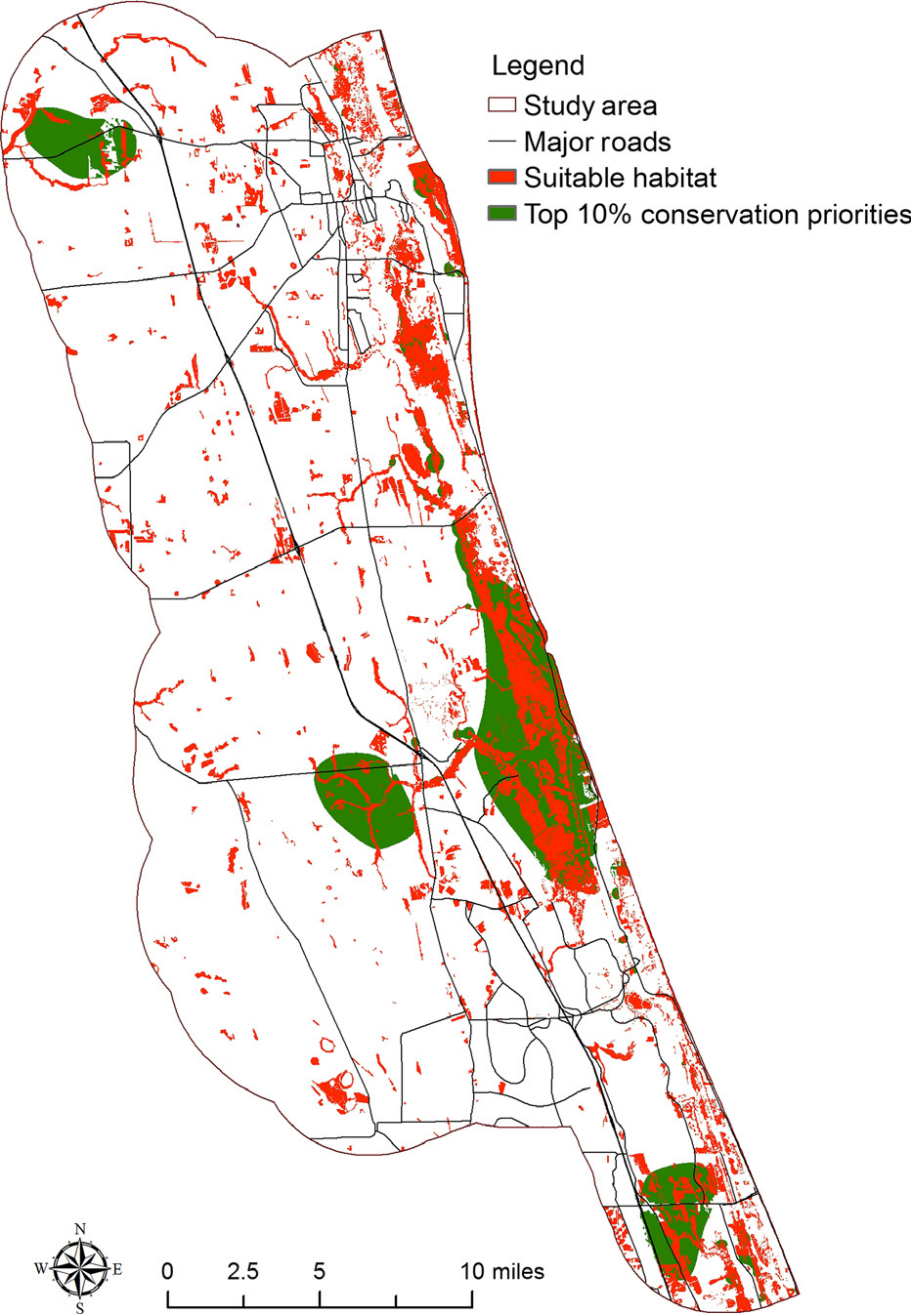
Comparison of the top 10% conservation priorities for the SLAMM–Lidar combination with suitable species habitat from the SLAMM–Lidar combination.

**Figure 8:**
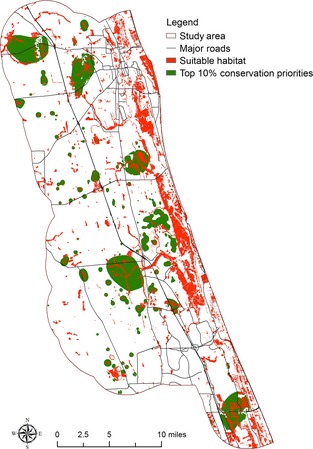
Comparison of the top 10% conservation priorities for the bathtub‐Lidar combination with suitable species habitat from the SLAMM–Lidar combination.

**Figure 9:**
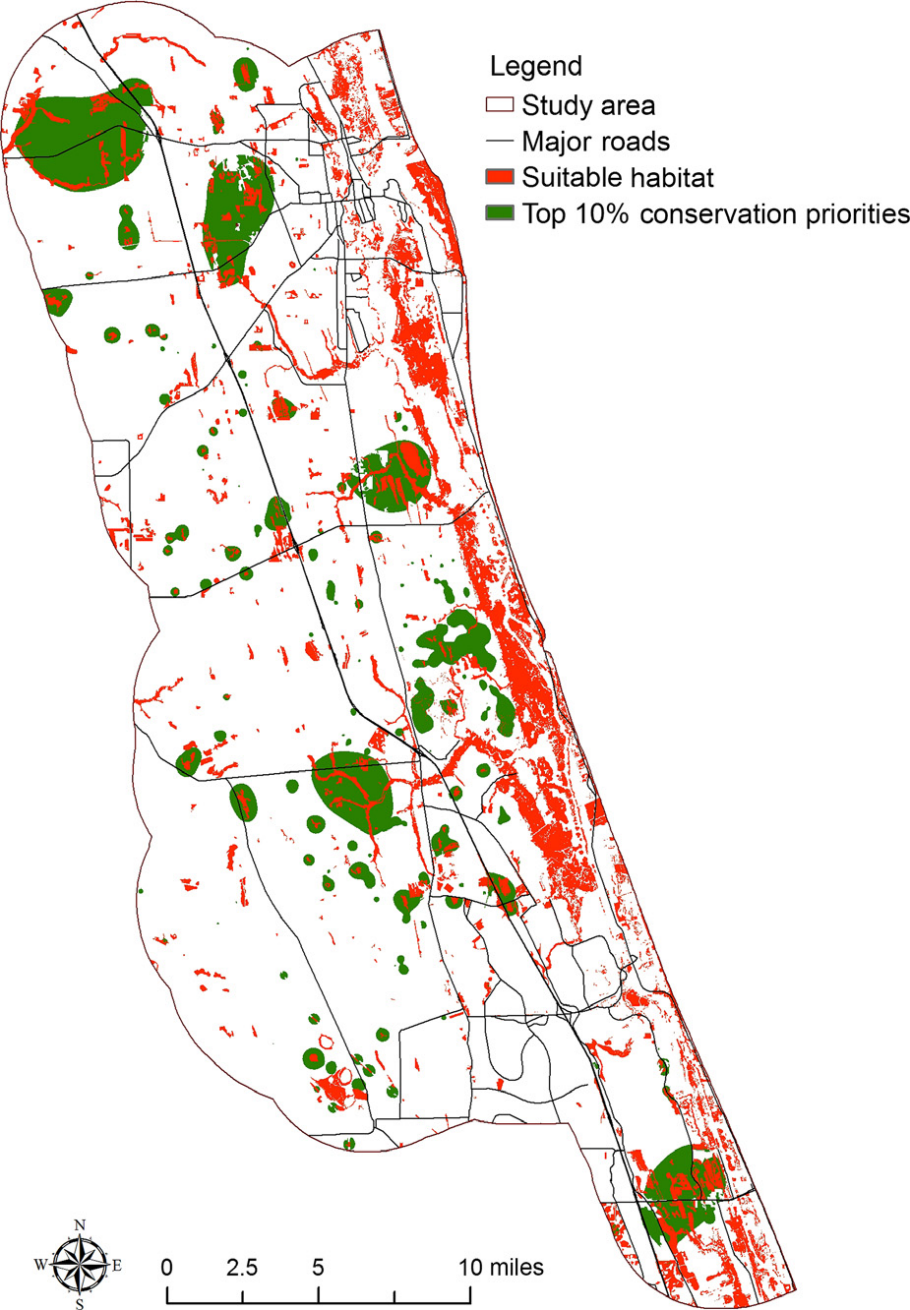
Comparison of the top 10% conservation priorities for the bathtub‐NED combination with suitable species habitat from the SLAMM–Lidar combination.

Four conservation prioritization scenarios including current conservation prioritization and conservation prioritization under the 1.0 m SLR scenario with different model–dataset combinations were identified (Fig. 5). Using conservation outcomes from the SLAMM–Lidar combination as benchmark, it can be seen that a large proportion of the top conservation priorities identified by the SLAMM–Lidar combination is not identified by the Bathtub–NED and Bathtub–Lidar combinations. Figure 6 shows the differences of top 10% conservation priorities in these scenarios. Compared to the top 10% conservation priorities identified by the SLAMM–Lidar combination, the Bathtub–NED combination excludes 17,815 acres of land and the Bathtub–Lidar combination excludes 16,433 acres of lands included within the top 10% conservation priorities by the SLAMM–Lidar combination. Figures 7, 8, 9 show the comparison of the top 10% conservation priorities for each model–dataset combination with suitable species habitat from the SLAMM–Lidar combination. Results indicate that using the bathtub model could lose a large number of suitable habitats as top conservation priorities (Table 4). The results indicate that different model–dataset combinations could lead to significant differences in conservation prioritization outcomes and the less accurate model and dataset could fail to identify areas of high conservation value that are important for adaptation to SLR identified by the SLAMM–Lidar combinations. The spatially explict analysis in this research could inform coastal reserve managers and other decision makers about the importance of sophisticated coastal impact model and high‐resolution elevation data in conservation planning for SLR.

**Table 4:** Top 10% conservation priorities for each model–dataset combination fall with suitable species habitat from the SLAMM–Lidar combination

	Bathtub–NED	Bathtub–Lidar	SLAMM–Lidar
Top 10% conservation priorities within suitable habitat identified by the SLAMM–Lidar combination	5081.02	4883.59	11,172.45

## Discussion

It has been recognized that we should use better data and coastal impact models to assess SLR impacts (Poulter and Halpin 2008; Gesch 2009). Nevertheless, this does not mean that we should use the more accurate model and dataset under all situations. There is an increasing availability of Lidar elevation data in coastal areas of the United States as the 3DEP Project is being implemented (Snyder 2013; Sugarbaker et al. 2014). However, we should realize that in some data‐poor areas of the world this high quality elevation data is not always obtainable. In addition, running the more sophisticated coastal impact models such as SLAMM is costly and requires expert knowledge which may not be available or feasible when budgets are constrained. One of the advantages of using a bathtub model to assess SLR impacts is that it provides a straightforward and easy to understand picture of potential SLR impacts, while also generally showing a larger geographic area inundated which may be appropriate for some planning situations. However, the disadvantages of using a bathtub model include its lack of simulation of wetlands and shoreline modifications due to SLR, thus could potentially underestimate suitable habitat for some marsh‐dependent species.

Choosing which model to be used in SLR impact assessment depends on the goal of the planning or research. We recommend using the most sophisticated model and high‐resolution elevation dataset available for identification of conservation priorities in response to SLR, where accuracy directly impacts the type and distribution of priorities that are identified. However, the bathtub model is useful for coarse SLR vulnerability assessments where budget, technical expertise, or data availability are issues, or where a more conservative and/or simple result is most ideal. Bathtub‐based assessments, including ones using coarser DEM data, can still be useful when better data and models are not available or feasible, but results need to be appropriately assessed and communicated.

The comparison of conservation outcomes using different model–dataset combinations in reserve design demonstrated the value of more detailed information (model sophistication and data accuracy) in SLR adaptive conservation planning and reserve design. Because of timing and funding, this research only compared conservation outcomes with three different model–dataset combinations including Bathtub–NED, Bathtub–Lidar, and SLAMM–Lidar, but the SLAMM–NED combination was not included in the analysis. Including the SLAMM–NED combination in the analysis would add to the existing research and present the conservation outcomes with a complex coastal impact model and coarse elevation data. Compared to conservation outcomes with the SLAMM–Lidar combination, this added analysis would likely demonstrate the value of more detailed elevation data in identifying SLR adaptive conservation priorities while using the complex coastal impact model. Including SLAMM–NED combination in the analysis would be a future research priority for assessing conservation outcomes with different model–dataset combinations. The conservation priorities presented here are purely ecological priority areas that economic concepts were not included in planning process. Given that economic costs could have a large impact on conservation outcomes (Naidoo et al. 2006), a future research priority is to identify conservation priorities under SLR while including consideration of conservation costs and compare the conservation outcomes with/without consideration of economic factors.

It is likely not necessary to include different SLR scenarios in the analysis as the aim of this study is to examine conservation outcomes of different model–dataset combinations although this analysis might be useful in the future because differences in predictions and identified priority areas might be significantly different for different SLR levels. Higher rates of SLR will lead to a greater change in vegetation distribution so the bathtub model might have a greater chance of incorrectly predicting this distribution (Runting et al. 2013), and lower rates of SLR would likely result in less difference between the model–dataset combinations A future research priority is to investigate how conservation priorities may vary among different SLR scenarios including both higher and lower rates of SLR when different model–dataset combinations are used.

## Conflict of Interest

None declared.

## Crosswalk between SLAMM and Florida Land Use, Cover and Forms Classification System

**Table:**

SLAMM code	SLAMM description	FLUCCS code
1	Developed dry land	Not used
2	Undeveloped Dry Land	Not used
3	Swamp	6300
4	Cypress Swamp	6210
5	Inland Fresh Marsh	6410
6	Tidal Fresh Marsh	6410
7	Scrub Shrub	6120
8	Regularly Flooded Marsh	6420
9	Mangrove	6120
10	Estuarine Beach	6510
11	Tidal Flat	6510
12	Ocean Beach	7100
14	Rocky Intertidal	6540
15	Inland Open Water	5000
17	Estuarine Water	5400
18	Tidal Creek	5400
19	Open Ocean	5700
20	Irregularly Flooded Marsh	6420
25	Vegetated Tidal Flat	6420

## References

[ece31669-bib-0001] Aiello‐Lammens, M. E. , M. Chu‐Agor , M. Convertino , R. A. Fischer , I. Linkov , and H. Resit Akcakaya . 2011 The impact of sea‐level rise on Snowy Plovers in Florida: integrating geomorphological, habitat, and metapopulation models. Glob. Change Biol. 17:3644–3654.

[ece31669-bib-0002] Callaway, J. C. , R. D. DeLaune , and W. H. Patrick Jr . 1997 Sediment accretion rates from four coastal wetlands along the Gulf of Mexico. J. Coastal Res., 13:181–191.

[ece31669-bib-0003] Church, J. A. , P. U. Clark , A. Cazenave , J. M. Gregory , S. Jevrejeva , A. Levermann , et al. 2013 Sea‐level rise by 2100. Science, 342:1445. 24357297 10.1126/science.342.6165.1445-a

[ece31669-bib-0004] Clough, J.S , R. A. Park , and R. Fuller . 2010 SLAMM 6 beta technical documentation. Warren Pinnacle Available at http://warrenpinnacle.com/prof/SLAMM6/SLAMM6_Technical_Documentation.pdf. (accessed 5 November 2012).

[ece31669-bib-0005] Cooper, H. M. , Q. Chen , C. H. Fletcher , and M. M. Barbee . 2013 Assessing vulnerability due to sea‐level rise in Maui, Hawai'i using LiDAR remote sensing and GIS. Clim. Change. 116:547–563.

[ece31669-bib-0006] Cox, J. 1994 Closing the gaps in Florida's wildlife habitat conservation system: recommendations to meet minimum conservation goals for declining wildlife species and rare plant and animal communities. Office of Environmental Services, FG & FWFC, Tallahassee, FL.

[ece31669-bib-0007] Cox, J. A. , and R. S. Kautz . 2000 Habitat conservation needs of rare and imperiled wildlife in Florida. Office of Environmental Services, Florida Fish and Wildlife Conservation Commission, Tallahassee, FL.

[ece31669-bib-0008] Craft, C. , J. Clough , J. Ehman , S. Joye , R. Park , S. Pennings , et al. 2008 Forecasting the effects of accelerated sea‐level rise on tidal marsh ecosystem services. Front. Ecol. Environ. 7:73–78.

[ece31669-bib-0009] Gesch, D. B. 2007 The National Elevation Dataset Pp. 99–118 *in* Maune D., ed. Digital elevation model technologies and applications: the DEM users manual, 2nd ed. American Society for Photogrammetry and Remote Sensing, Bethesda, MD.

[ece31669-bib-0010] Gesch, D. B . 2009 Analysis of lidar elevation data for improved identification and delineation of lands vulnerable to sea‐level rise. J. Coastal Res. 53:49–58.

[ece31669-bib-0011] Gesch, D. B. 2013 Consideration of vertical uncertainty in elevation‐based sea‐level rise assessments: Mobile Bay, Alabama case study. J. Coastal Res. 63(sp1):197–210.

[ece31669-bib-0012] Hoctor, T. S. 2011 Impacts Assessment Decision Support Model Project for the Southwest Florida Water Management District, Final Report. Southwest Florida Water Management District, Brooksville, FL.

[ece31669-bib-0013] Kinser, P. , D. Curtis , B. Beck , and C. Yates . 2007 Coastal wetlands of the Guana‐Tolomato‐Matanzas National Estuarine Research Reserve in Northeast Florida. St. Johns Water Management District, Palatka, FL.

[ece31669-bib-0014] Knight, G. R. , F. N. A. I. Director , D. Hipes , A. Jenkins , C. Elam , P. Diamond , et al. 2010 Development of a Cooperative Land Cover Map: Final Report 15 July 2010. Available at http://www.fnai.org/PDF/Cooperative_Land_Cover_Map_Final_Report_20101004.pdf. (Accessed July 5th, 2014).

[ece31669-bib-0015] Lauro, B. , and J. Burger . 1989 Nest‐site selection of American Oystercatchers (*Haematopus palliatus*) in salt marshes. Auk 106:185–192.

[ece31669-bib-0016] Linhoss, A. C. , G. Kiker , M. Shirley , and K. Frank . 2015 Sea‐level rise, inundation, and marsh migration: simulating impacts on developed lands and environmental systems. J. Coastal Res. 31:36–46.

[ece31669-bib-0017] Lowther, P. E. , S. M. Lanyon , and C. W. Thompson . 1999 Painted Bunting(*Passerina ciris*). Birds North America 398:24.

[ece31669-bib-0018] Mcleod, E. , B. Poulter , J. Hinkel , E. Reyes , and R. Salm . 2010 Sea‐level rise impact models and environmental conservation: a review of models and their applications. Ocean Coast. Manag. 53:507–517.

[ece31669-bib-0019] Moilanen, A. , A. M. Franco , R. I. Early , R. Fox , B. Wintle , and C. D. Thomas . 2005 Prioritizing multiple‐use landscapes for conservation: methods for large multi‐species planning problems. Proc. Biol. Sci. 272:1885–1891. 16191593 10.1098/rspb.2005.3164 PMC1559892

[ece31669-bib-0020] Moilanen, A. , H. Kujala , and J. R. Leathwick . 2009 The Zonation framework and software for conservation prioritization Pp. 196–210 *in* Moilanen Atte, wilson Kerrie A. and Possingham Hugh P., eds. Spatial conservation prioritization. Oxford University Press, New York.

[ece31669-bib-0021] Moilanen, A. , L. Meller , J. Leppanen , F.M. Pouzols , A. Arponen , and H. Kujala . 2012 Spatial conservation planning framework and software Zonation v3.1 user manual. Helsinki, Finland Available at http://cbig.it.helsinki.fi/software/zonation/. (accessed 25 June 2013).

[ece31669-bib-0022] Naidoo, R. , A. Balmford , P. J. Ferraro , S. Polasky , T. H. Ricketts , and M. Rouget . 2006 Integrating economic costs into conservation planning. Trends Ecol. Evol. 21:681–687. 17050033 10.1016/j.tree.2006.10.003

[ece31669-bib-0023] Noss, R. F. 2011 Between the devil and the deep blue sea: Florida's unenviable position with respect to sea level rise. Clim. Change. 107:1–16.

[ece31669-bib-0024] NWF . 2006 An unfavorable tide – global warming, coastal habitats and sport fishing in Florida. National Wildlife Federation, Florida Wildlife Federation, Merrifield, VA.

[ece31669-bib-0025] Poulter, B. , and P. N. Halpin . 2008 Raster modelling of coastal flooding from sea‐level rise. Int. J. Geogr. Inf. Sci. 22:167–182.

[ece31669-bib-0026] Rogers, C. M. , M. J. Taitt , J. N. Smith , and G. Jongejan . 1997 Nest predation and cowbird parasitism create a demographic sink in wetland‐breeding Song Sparrows. Condor 99:622–633.

[ece31669-bib-0027] Runting, R. K. , K. A. Wilson , and J. R. Rhodes . 2013 Does more mean less? The value of information for conservation planning under sea level rise. Glob. Change Biol. 19:352–363. 10.1111/gcb.12064 23504775

[ece31669-bib-0400] Snyder, G. I. 2013 The benefits of improved national elevation data. Photogramm. Eng. Remote Sens 79:105–110.

[ece31669-bib-0401] Sugarbaker, L. J. , E. W. Constance , H. K. Heidemann , A. L. Jason , V. Lukas , D. L. Saghy , et al. 2014 The 3D Elevation Program initiative: a call for action. US Geological Survey 1399. 12–13.

